# Effectiveness of physical therapy treatment in addition to usual podiatry management of plantar heel pain: a randomized clinical trial

**DOI:** 10.1186/s12891-019-3009-y

**Published:** 2019-12-28

**Authors:** Shane M. McClinton, Bryan C. Heiderscheit, Thomas G. McPoil, Timothy W. Flynn

**Affiliations:** 1 Doctor of Physical Therapy Program, Des Moines University, 3200 Grand Avenue, Des Moines, IA USA; 2 Departments of Orthopedics & Rehabilitation and Biomedical Engineering, and Doctor of Physical Therapy Program, University of Wisconsin-Madison, 1636 Highland Ave, Madison, WI USA; 3 School of Physical Therapy, Regis University, Denver, CO USA; 4 School of Physical Therapy, South College, Knoxville, TN USA

**Keywords:** Exercise, Manual therapy, Physiotherapy, Plantar fasciitis, Podiatrist

## Abstract

**Background:**

Many patients will seek care from a podiatrist for plantar heel pain (PHP), while few of these patients will also be seen by a physical therapist. Physical therapists can provide treatment that is not a part of routine podiatric care for PHP and may provide additional improvement. Therefore, the purpose of this study was to examine the effects of interdisciplinary care for PHP that incorporated physical therapy treatment after initiating podiatric treatment.

**Methods:**

Eligible individuals with PHP that presented to a podiatrist were randomized to receive usual podiatric care (uPOD) or usual podiatric care plus physical therapy treatment (uPOD+PT). The primary outcome was change in foot and ankle ability measure (FAAM) at 6-months. Secondary outcomes included change in numeric pain rating scale (NPRS), patient-reported success, and 6-week and 1-year endpoints. Patient-reported success was defined as the top two global rating of change scale rankings. Primary analysis was intention-to-treat (ITT) using analysis by covariance adjusted to baseline scores, and a secondary per-protocol (PP) analysis was performed analyzing only those who completed treatment.

**Results:**

Ninety-five individuals participated and were included in the ITT analysis, and 79 were included in the PP analysis. For the primary outcome of FAAM change from baseline to 6-months, both groups improved significantly (uPOD+PT: 26.8 [95% CI 21.6, 31.9]; uPOD: (20 [15.6, 24.4]), but there was no between-group difference (4.3 [− 1, 9.6]). For secondary outcomes, the uPOD+PT group demonstrated greater improvement in NPRS at 6 weeks (0.9 [0.3, 1.4]) and 1 year (1.5 [0.6, 2.5]) in the ITT analysis. In the PP analysis, the uPOD+PT group demonstrated greater improvement in FAAM at 6 months (7.7 [2.1, 13.3]) and 1 year (5.5 [0.1, 10.8]), NPRS at 6 weeks (0.9 [0.2, 1.6]), 6 months (1.3 [0.6, 2.1]) and 1 year (1.3 [0.6, 2.1]), and in patient-reported success (relative risk [95% CI]) at 6 weeks (2.8 [1.1, 7.1]), 6 months (1.5 [1.1, 2.1]), and 1 year (1.5 [1.1, 1.9]).

**Conclusions:**

There was no significant benefit of uPOD+PT in the primary outcome of FAAM change at 6 months. Secondary outcomes and PP analysis indicated additional benefit of uPOD+PT, mostly observed in individuals who completed treatment.

**Trial registration:**

Prospectively registered May 24, 2013 at www.clinicaltrials.gov (NCT01865734).

## Background

Plantar heel pain (PHP) is a common foot condition managed by various healthcare providers including podiatrists, physical therapists, primary care physicians, orthopaedic surgeons, and chiropractors. Plantar fasciitis is a term commonly used for PHP, but the term PHP comprises the various pathoanatomical features of this condition to include plantar fascia inflammation, degeneration or thickening, heel fat pad pathology, nerve irritation, and heel spurs [1–4]. The prevalence of PHP in adults is between 3.6–9.6% [5–8] and physicians treat 1–2 million Americans with PHP annually at a cost of $192 -$376 million [9, 10]. The burden of management is likely larger if all providers involved in PHP care are included in this estimate.

There are many approaches described to treat PHP, but there is no strong evidence that any of the most common treatments (exercise [mostly stretching of the plantar fascia, calf and Achilles tendon], non-steroidal anti-inflammatory drugs, corticosteroid injections, foot orthoses, extracorporal shockwave therapy) are superior to another [11]. Other treatments described to manage PHP include wearing supportive footwear, foot taping, manipulation/mobilization of the foot, ankle, and lower leg, foot and lower leg strengthening, and platelet-rich plasma injections [2, 12–18]. Consistent with these treatments that are local to the painful area, many impairments associated with PHP include altered foot posture/mobility, limited ankle or hallux dorsiflexion, increased daily weightbearing, lower leg/foot muscle performance, and inflammation, thickening or degeneration of the plantar fascia [1, 2, 4, 19–23]. A local treatment approach for PHP is more effective than placebo or no treatment [11], and 80–90% of individuals recover or are satisfied with their outcome at 1 year following localized treatments [24–26]. However, outcomes at 8 weeks to 4 years following treatment indicate that many will have residual symptoms and the potential for persisting disability and recurrence of PHP [25–28]. Likelihood of residual symptoms lessens over time; for example, outcome studies indicate 83% of patients were less than 75% recovered at 8 weeks [29], 49% of patients had symptoms at 6 months [26], 24% of patients felt the need for further care at 2 years [27], and 18% had continued symptoms that limited function at 4 years following treatment [25]. In individuals who initially recover from PHP, 30% will have a recurrence of symptoms [28].

Lack of long-term recovery in many individuals with PHP indicates the need to improve current management strategies. Emerging considerations in the management of PHP include treatment efficacy of manual therapy and evidence of the contribution of impairments more proximal on the body associated with PHP. Manual therapy to the calf, foot, ankle, knee, and hip has demonstrated clinically-meaningful improvements in pain and function [15, 30]. In addition, impairments in the knee, posterior thigh, hip, low back, and the peripheral and central nervous system have been linked to PHP or foot and ankle problems [31–37]. Because physical therapists can perform manual therapy interventions in addition to assessment and treatment of both local and proximal impairments that may contribute to PHP, physical therapists may add benefit to the usual management of PHP of other primary care providers, such as podiatrists. Plantar heel pain is the most prevalent condition seen in podiatry practice constituting up to 10–15% of all patients [38, 39], and in the United States, only 7.1% of patients seeking care for PHP are seen by a physical therapist within 30 days of the initial diagnosis [40]. Therefore, the purpose of this study was to examine the effects of interdisciplinary care for PHP that incorporated physical therapy treatment after initiating podiatric treatment.

## Methods

### Study design

This study was a parallel-group randomized pragmatic clinical trial with outcomes assessed at 6 weeks, 6 months, and 1 year following the initial visit with a podiatrist. Institutional Review Board approval was received from the Des Moines University and Rocky Mountain University of Health Professions Review Boards. Verbal and written consent to participate in the clinical trial was obtained from each participant prior to the baseline assessment and the rights of participants were protected. The trial was registered prospectively (ClinicalTrials.gov NCT01865734) and the study protocol was published with open access [41].

### Participants

Individuals between the ages of 18–70 years with a primary complaint of PHP that presented to the Podiatry Department of the Des Moines University Clinic between March 2014 through November 2016 were recruited. For the first 5 months of the trial, individuals over the age of 60 were excluded; the age restriction was increased to 70 after preliminary analysis of recruitment and to improve generalizability to the typical clinic population. Eligible participants were diagnosed with PHP by a podiatrist based on characteristics including tenderness to palpation at, or near the plantar fascia insertion site and pain associated with the first step after waking in the morning or with progression of weight-bearing during the day. Participants were excluded if they had a foot and ankle ability measure activities of daily living subscale (FAAM) score greater than 88/100, symptoms for longer than 1 year, surgery of the foot, ankle or lower leg, clinical signs of radiculopathy, contraindications to manual therapy interventions (i.e., tumor, fracture, rheumatic inflammatory disease [rheumatoid, reactive and psoriatic arthritis, inflammatory bowel disease, ankylosing spondylitis, and systemic lupus erythematosus], osteoporosis, prolonged history of steroid use, severe vascular disease, etc.), clinical indication of plantar fascia rupture, received prior treatment for PHP in the past 6 weeks, or were unable or unwilling to complete questionnaires or comply with treatment recommendations of either treatment group. Initially, participants with a BMI > 30 kg/m^2^ were to be excluded [41], but after trial commencement and before enrollment, the study protocol was amended by removing the BMI exclusion criterion following further evidence that a high BMI was not a predictor of clinical success in response to physical therapy treatment for PHP [42].

### Outcome assessments

Prior to randomization, all participants completed one visit with a podiatrist where usual care was provided and a baseline assessment that included all primary and secondary outcomes and demographic information. The 6-week, 6-month, and 1-year assessments were completed independently by participants and returned to the investigative team via postal mail, email, fax, or delivered to the clinic’s office personnel. The primary outcome was the 6-month outcome of the FAAM activities of daily living subscale which is a 21-item questionnaire scored from 0 to 100 where 0 represents an inability to perform daily activities because of the feet and ankles and 100 indicates no difficulty. The FAAM has been examined in individuals with lower leg, foot, and ankle-related disorders (approximately 10% with PHP) and demonstrated high content and construct validity, has a test-retest reliability equal to ICC = 0.89, and an MCID of 8 points [43]. Secondary outcomes included the FAAM at 6 weeks and 1 year, the numeric pain rating scale (NPRS) and the global rating of change (GRC) measured at 6 weeks, 6 months, and 1 year. A 3-item NPRS was used that rated the current, best, and worst levels of pain over the prior week and has test-retest reliability equal to ICC = 0.61 (95% confidence interval [CI]: 0.3, 0.77) and minimal clinically important difference (MCID) of 2 points [44, 45]. The GRC scale used ranked improvement from − 7 (a very great deal worse) to 0 (about the same) to + 7 (a very great deal better) [46]. Scores equal to or greater than + 6 (a great deal better) were used as an indicator of clinical success [47]. Adherence to intervention was recalled by participants at each follow-up using a numeric rating scale where 0 = no treatment completed and 10 = completed all treatment as instructed. Categories of treatment that participants were asked to self-assess included medication, foot orthosis, exercises recommended by podiatrist, exercises recommended by physical therapist, footwear modifications, and activity modifications. Participants were asked to report any issue or injuries associated with treatment when completing follow-up questionnaires to assess adverse events related to treatment.

### Randomization

Following the baseline assessment, participants were randomized to usual podiatric care (uPOD) or uPOD plus physical therapy treatment (uPOD+PT) following a computer-generated randomization list with varying block sizes of four and six. The allocation sequence was concealed from the investigator enrolling participants by sequentially numbered, opaque sealed envelopes [48]. The envelopes were only accessible to the principal investigator who enrolled participants and performed allocation after baseline assessment was completed by documenting the participant identification number, signing, and dating the envelop prior to opening [48].

### Interventions

All participants in this investigation were seeking care from a podiatrist for their PHP and therefore, all participants completed at least one visit with a podiatrist where an evaluation was performed to confirm the diagnosis and intervention provided at the discretion of the podiatrist. The podiatrist provided education about the diagnosis, recommended wearing supportive shoes as much as possible, prescribed medication and/or foot orthoses when warranted (Additional file 1), provided a handout that emphasized calf and plantar foot stretches, and had the option to refer patients to a physical therapist or to order further imaging. Following this appointment, participants received either uPOD or uPOD+PT depending upon random allocation. Five podiatrists from the Des Moines University Foot and Ankle Clinic provided uPOD treatment. All podiatrists were Fellows in the American College of Foot and Ankle Surgeons with nine to 38 years of clinical experience treating PHP. Three physical therapists from the Des Moines University Physical Therapy Clinic provided physical therapy treatment. The physical therapists had 13 to18 years of clinical experience treating PHP and one therapist was board certified in orthopaedic physical therapy and a Fellow of the American Academy of Orthopaedic Manual Physical Therapists.

#### Usual podiatric care (uPOD)

Podiatry treatment was performed in accordance with usual practice patterns of the providers at the Foot and Ankle Clinic of the Des Moines University Clinic. The number and frequency of follow-up visits with the podiatrist and the volume of podiatry interventions indicated by the units of current procedural terminology (CPT) and healthcare common procedure coding system (HCPCS) codes billed is described in Table 1 and Additional file 1.

**Table 1:** Details of treatment provided to the usual podiatric care (uPOD) and usual podiatric care plus physical therapy treatment (uPOD+PT) groups. Values are median (IQR)

Treatment detail	uPOD+PT (*n* = 48)	uPOD (*n* = 47)
Number of total visits	6 (4–8)	2 (2–6)^a^
Number of visits with podiatrist	1 (1–2)	2 (1–3)^a^
Duration of visits with podiatrist (days)	1 (1–31)	29 (1–55)^a^
Number of visits with physical therapist	4 (2.3–5.8)	0 (0–1)^a,b^

^a^Significantly different from the uPOD+PT group, *P* < 0.04. ^b^Eleven participants in the uPOD group received physical therapy treatment during the study for a median of 6 (IQR 3–10) visits

#### Physical Therapy Treatment in Addition to Usual Podiatric care (uPOD + PT)

Physical therapy intervention was provided as an extension of care received from the podiatrist. In our protocol paper [41], this was referred to as early physical therapy intervention; but because timing of physical therapy intervention was not manipulated in this study, the term uPOD+PT was felt to be more appropriate and is used in this paper. Participants in the uPOD+PT group followed-up with their podiatrist as per the podiatrist’s recommendations and the physical therapist sent progress notes to the podiatrist prior to each follow-up appointment. The physical therapist and podiatrist discussed the care plan or any issues in person or via telephone on an as-needed basis. Physical therapy treatment was guided by the plantar heel pain practice guidelines from the Academy of Orthopaedic Physical Therapy (formerly known as the Orthopaedic Section of the American Physical Therapy Association) [2, 3] and a manual of procedures that directed intervention priorities and progression based on current best evidence. The lead author trained all treating physical therapists in the manual of procedures that involved a 3-phase progression. After initial training, therapists consulted the lead author as needed for clarification of treatment procedures or progression. All treating physical therapists and the lead author worked in the same clinic which supported consistency of treatment and assessment of treatment fidelity. Phase of treatment included: 1) symptom reduction, 2) graded exercise to restore muscle performance and gait, and 3) graded activity including return to sport or recreation [49]. Individual treatment was provided at the discretion of the physical therapist and was based on impairments and centered on the participant’s presentation, goals, and short-term treatment response. Participants were asked to initially attend clinic appointments 1 time per week, on average, and the time between appointments was lengthened as the patient demonstrated independence in self-directed management in later phases of treatment. Physical therapy interventions included a combination of manual therapy, patient education, stretching, resistance training, and neurodynamic interventions. Additional details of treatment phases, specific interventions and clinical reasoning common to the physical therapy treatment are described by McClinton et al. [49].

### Power and statistical analysis

Sample size for the primary outcome, FAAM at 6 months, was estimated using G*Power 3.1.5 based on detecting a difference between groups greater than the minimal clinically important difference (MCID) of 8 points in the FAAM with an alpha level of 0.05, 80% power, and pooled sample variance of 14.5 from Cleland et al. [43, 50, 51]. This resulted in an effect size of 0.62 and 42 participants needed per group. To account for loss to follow-up, 56 participants were initially planned to be recruited [41], but fewer participants were lost to follow-up than predicted. Therefore, enrollment was terminated by the principal investigator after enrollment of 47 participants per group when it was evident that the sample size determined a-priori was achieved.

Baseline group variables were summarized using the mean and standard deviation for continuous measures and percentages for categorical measures. Parametric test assumptions were analyzed for all continuous variables by visual inspection of histograms, use of skewness score within double the standard error of skewness criteria, and Levene’s test for homogeneity of the variance. Analysis of primary and secondary outcomes were conducted by intention-to-treat (ITT) with participants analyzed according to random assignment regardless of post-randomization exclusion status or adherence. Intention-to-treat analysis was performed instead of the original plan to use post-randomization exclusion by committee [41] to reduce bias in estimates of treatment effect and to be consistent with CONSORT guidelines [52]. Because of the change in post-randomization exclusion, a sensitivity analysis was performed to assess the influence of ITT methods compared to a per-protocol (PP) analysis that only included those who completed treatment. In both groups, participants were considered to complete treatment if they attended clinic appointments or follow-up according to the plan mutually set by the participant and provider as indicated in the participant’s medical record [53–55]. In addition, for participants assigned to the uPOD+PT group, they were considered to complete treatment if they attended at least 4 visits with a physical therapist regardless of adherence to recommended follow-up appointments. Participants that did not complete treatment failed to follow-up as planned and, in the uPOD+PT group, attended fewer than 4 visits. The completer criteria of 4 visits in the uPOD+PT group was used to represent the average number of visits with a physical therapist for PHP of 4.9–5.1 [40, 56], to assure that care continuity was established [57], to reduce the potential for diminished response with fewer than 4 visits [58], and to reduce the likelihood of removing participants that did not follow-up due to lack of response to treatment. The completer criteria were determined prior to any data analysis. Multiple imputation by chained equations was used for missing data. The mice package of R (R Foundation for Statistical Computing, Vienna, AT) was used to create 10 imputed data sets using available primary and secondary outcomes, age, weight, height, BMI, health quality (EQ-5D [59]), symptom duration, education level, and smoking status [60]. Separate analyses of covariance were used to compare group differences in the FAAM and NPRS change from baseline to the 6-week, 6-month, and 1-year follow-up controlling for baseline scores. Pairwise comparisons were also performed to examine within-group changes at each follow-up. The χ^2^ test was used to compare proportions of participants in each group that reported success with treatment and that improved greater than, or equal to the MCID for the NPRS (2 points [44, 45]) and FAAM (8 points [43]). Success was defined as the top two ratings on the GRC scale indicating the participant perceived they were “a great deal better” or “a very great deal better.” The Mann-Whitney Test was used to compare between-group differences in the number of visits, duration of treatment, time to the first appointment with a PT, procedures, and adherence. All analyses except for multiple imputation were performed using SPSS Version 22 (IBM Corporation, Armonk, NY).

### Blinding

Patient-reported outcomes including the FAAM, NPRS, and GRC scale were completed by the patients independently. Questionnaires were collected by investigators that were not blinded to treatment group, but outcome scores were calculated and data entered into a spreadsheet by an individual blinded to treatment group. The dataset was blinded for statistical analysis and only unblinded once completed. Determination of treatment completion was made by the treating provider using only treatment plan details extracted from the medical record without knowledge of the participant or the participant’s outcome.

## Results

**Fig. 1:**
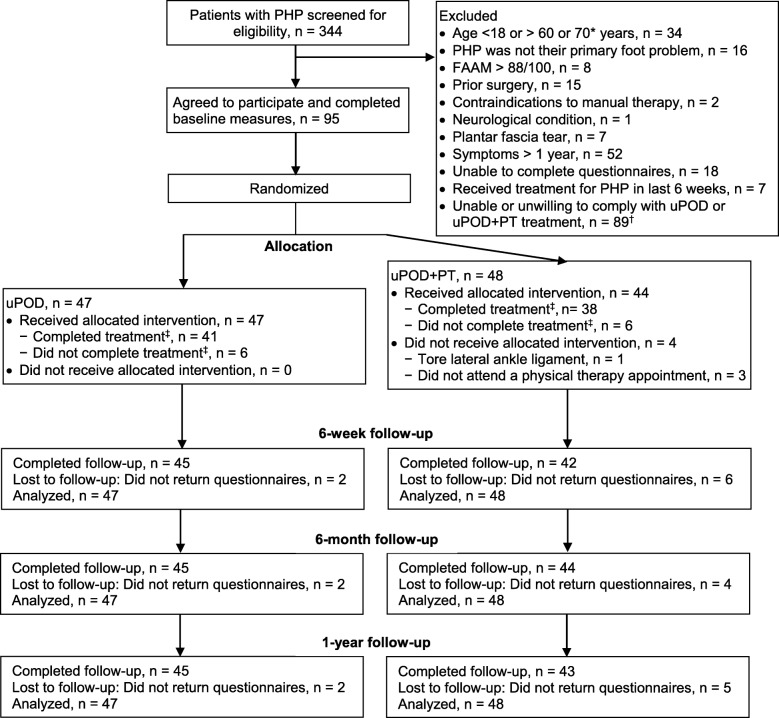
Flow diagram of participant recruitment and retention. *Upper age limit was 60 years for the first 5 months of enrollment and increased to 70 for the next 32 months until enrollment ended. ^†^Reasons provided were because the patient did not live near the clinic to attend regular appointments (*n* = 23), did not have time to participate (*n* = 27), was concerned about additional copayments or treatment-related costs (*n* = 19), or chose not to participate (*n* = 20). ^‡^Completers were defined as individuals who attended clinic appointments or follow-up according to the plan mutually set by the patient and provider as indicated in the participant’s medical record or completed at least 4 visits with a physical therapist if assigned to the usual podiatric care plus physical therapy treatment (uPOD+PT) group [53–55]. Individuals that did not complete treatment were participants who failed to follow-up as planned. PHP, plantar heel pain; uPOD, usual podiatric care; FAAM, foot and ankle ability measure

Ninety-five individuals agreed to participate and were randomized to uPOD+PT or uPOD. The participant flow diagram with details of numbers screened, excluded, randomized, completed follow-up, and analyzed is detailed in Fig. 1 and participant characteristics per group is in Table 2. Participant characteristics were similar between groups, although 6 more participants in the uPOD group had a prior history of PHP. Participants in the uPOD+PT group were seen for a median of 6 visits with a podiatrist or a physical therapist compared to 2 visits with a podiatrist in the uPOD group (Table 1). The volume of physical therapy and podiatry interventions received by participants indicated by the units of current procedural terminology (CPT) and healthcare common procedure coding system (HCPCS) codes billed is described in Additional file 1. Frequency of foot orthosis prescription and adherence and medication prescription and adherence were similar between groups (Additional files 1 and 2). Podiatry-specific procedures were similar between groups (Additional file 1) except that participants in the uPOD group received more established visits with a podiatrist. There were no adverse events reported by participants in either treatment group.

**Table 2:** Characteristics of all participants and participants that completed treatment in each group. Values are mean ± SD or frequency count (%)

Characteristic	uPOD+PT (*n* = 48)	Completed uPOD+PT (*n* = 38)	uPOD (*n* = 47)	Completed uPOD (*n* = 41)
Age (years)	49.8 ± 10.8	51 ± 11	50.3 ± 10.3	50.9 ± 10.1
Women	38 (79.2)	30 (78.9)	33 (70.2)	29 (70.7)
Height (cm)	170.1 ± 8.8	169. ± 9.4	170.6 ± 8.7	170.5 ± 8.4
Weight (kg)	92.6 ± 24.1	88.9 ± 18.8	91.1 ± 21.5	92.6 ± 21.7
BMI (kg/m^2^)	32 ± 7.6	31 ± 6.8	31.3 ± 6.9	31.8 ± 7.1
Prior history of PHP	14 (29.8)	11 (29.7)	20 (42.6)	17 (41.5)
Bilateral symptoms	12 (25)	8 (21.1)	15 (31.9)	12 (29.3)
Duration of symptoms (days)	129.2 ± 105.9	126.4 ± 111	147.2 ± 111.1	137.3 ± 97.7
Foot Posture Index (R, L)^a^	3.2 ± 3.2, 4 ± 3.4	3.4 ± 3.1, 4.2 ± 3.4	3.6 ± 3.3, 4.4 ± 3.2	3.6 ± 3.4, 4.4 ± 3.1
Number of hours on feet/day	7 ± 4.1	6.8 ± 4.1	6.4 ± 3.2	6.1 ± 3.1
NPRS	5.2 ± 1.9	5.3 ± 2	4.9 ± 1.8	4.9 ± 1.8
FAAM	61.6 ± 17.5	60.9 ± 17.8	65.3 ± 13	65 ± 13
Elevated fear avoidance beliefs^b^	32 (66.7)	26 (68.4)	29 (61.7)	26 (63.4)
General 6-month recovery expectation^c^	5.6 ± 1.9	5.8 ± 1.7	5.8 ± 1.7	5.8 ± 1.8

*BMI* body mass index, *FAAM* Foot and Ankle Ability Measure, *NPRS* numeric pain rating scale, *PHP* Plantar heel pain, *uPOD* usual podiatric care, *uPOD+PT* physical therapy treatment in addition to usual podiatric care. ^a^Foot posture category as defined by Redmond [71], normal = 0–5, pronated = 6–9, highly pronated = 10+, supinated = ^−^ 1-^−^ 4, highly supinated; ^b^Fear avoidance was rated on a Likert scale where 0 = completely disagree and 4 = completely agree to the physical activity screening question, “I should not do physical activities which (might) make my pain worse.” Scores greater than or equal to 2/4 indicate elevated fear avoidance beliefs [72]; ^c^Measured by rating expected recovery according to the Global Rating of Change scale where 5 = “quite a bit better” and 6 = “a great deal better” [46]

Over the study period 17 participants (uPOD = 12) were referred to a physical therapist and the time to referral ranged between 19 to 169 days. Eleven of 12 participants in the uPOD group referred to a physical therapist attended at least 1 visit with a physical therapist and, when compared to participants in the uPOD+PT group, had more median number of visits with a physical therapist (uPOD = 6 [IQR 3–10], uPOD+PT = 4 [IQR 2.3–5.8]), a greater median number of days until the first visit with a physical therapist (uPOD = 24.5 [IQR 13.5–43.8], uPOD+PT = 14.5 [IQR 7–27]), and longer median duration of physical therapy visits (uPOD = 60 [IQR 7.3–125.5], uPOD+PT = 33 [20.1–61]). Seven participants (uPOD = 4) were referred to a physical therapist at the initial visit with a podiatrist and before randomization, and 10 participants (uPOD = 6) were referred within 30 days of the initial visit with the podiatrist.

### Intention-to-treat analyses

#### Primary outcome

**Fig. 2:**
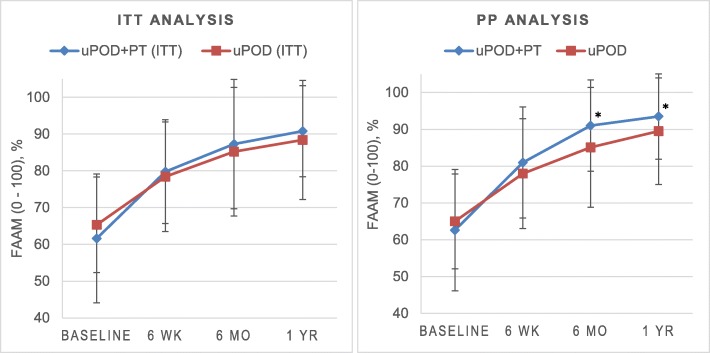
Foot and ankle ability measure (FAAM) at each assessment for the primary intention-to-treat (ITT) and per-protocol (PP) analyses. Higher scores indicate higher function on the FAAM. *Change in FAAM score from baseline significantly greater with usual podiatric care plus physical therapy treatment (uPOD+PT) versus usual podiatric care (uPOD) (*P* < 0.05)

There were no significant differences between uPOD+PT and uPOD in change from baseline in the primary outcome (FAAM) at 6 months (Table 3 and Fig. 2).

**Table 3:** Functional status and pain intensity outcomes of treatment for plantar heel pain. Values are mean ± SD or absolute mean difference (95% CI) for the intention-to-treat (ITT) and the per-protocol (PP) analyses comparing usual podiatric care (uPOD) and usual podiatric care plus physical therapy treatment (uPOD+PT)

	uPOD+PT		uPOD		Between-group difference in change from baseline^a^	*P*-value	Effect size (Cohen’s d)
	Mean ± SD	Within-group change from baseline	Mean ± SD	Within-group change from baseline			
FAAM (0–100)^b^							
ITT analysis (uPOD+PT, *n* = 48; uPOD, *n* = 47)							
Baseline	61.6 ± 17.5		65.3 ± 13				
6 wk	79.8 ± 14.1	18.2 (13.4 to 23.1)^d^	78.4 ± 14.9	13.1 (9.5 to 16.8)^d^	3.5 (− 1.6 to 8.5)	0.177	0.349
6 mo	87.3 ± 17.6	25.7 (20.3 to 31.1)^d^	85.2 ± 17.5	20 (15.5 to 24.3)^d^	4.5 (− 1.7 to 10.8)	0.153	0.345
1 yr	90.8 ± 12.4	29.3 (23.7 to 34.9)^d^	88.4 ± 16.2	23.2 (19.2 to 27.1)^d^	4.3 (−1.0 to 9.6)	0.113	0.368
PP analysis (uPOD+PT, *n* = 38; uPOD, *n* = 41)							
Baseline	62.6 ± 16.5		65 ± 13				
6 wk	81 ± 15.1	20.1 (14.4 to 25.7)^d^	78 ± 14.9	13 (9.1 to 16.9)^d^	5.1 (−0.7 to 11.0)	0.084	0.469
6 mo	91 ± 12.4	30.1 (25.6 to 34.6)^d^	85.1 ± 16.3	20.2 (15.4 to 25.0)^d^	7.7 (2.1 to 13.3)^e^	0.008	0.684
1 yr	93.5 ± 11.6	32.6 (27.0 to 38.2)^d^	89.5 ± 14.5	24.6 (20.6 to 28.5)^d^	5.5 (0.1 to 10.8)^e^	0.045	0.533
NPRS (0–10)^c^							
ITT analysis (uPOD+PT, *n* = 48; uPOD, *n* = 47)							
Baseline	5.2 ± 1.9		4.9 ± 1.8				
6 wk	2.7 ± 1.5	2.5 (1.9 to 3.1)^d^	3.4 ± 1.6	1.5 (1.0 to 1.9)^d^	0.9 (0.3 to 1.4)^e^	0.003	0.583
6 mo	1.9 ± 1.8	3.3 (2.5 to 4.0)^d^	2.6 ± 2.1	2.3 (1.7 to 2.9)^d^	0.7 (−0.05 to 1.5)	0.067	0.412
1 yr	1.1 ± 1.3	4.1 (3.5 to 4.8)^d^	2.1 ± 2.3	2.8 (2.1 to 3.5)^d^	1.5 (0.6 to 2.5)^d^	0.001	0.557
PP analysis (uPOD+PT, *n* = 38; uPOD, *n* = 41)							
Baseline	5.2 ± 2		4.9 ± 1.8				
6 wk	2.7 ± 1.6	2.6 (1.9 to 3.3)^d^	3.5 ± 1.6	1.5 (1.0 to 2.0)^d^	0.9 (0.2 to 1.6)^e^	0.008	0.617
6 mo	1.3 ± 1.4	4 (3.2 to 4.7)^d^	2.6 ± 2	2.4 (1.7 to 3.0)^d^	1.3 (0.6 to 2.1)^d^	0.001	0.736
1 yr	0.7 ± 0.9	4.6 (3.9 to 5.3)^d^	2 ± 2.2	3 (2.2 to 3.7)^d^	1.3 (0.6 to 2.1)^d^	0.001	0.702

*FAAM* foot and ankle ability measure, *NPRS* numeric pain rating scale. ^a^Adjusted for baseline score; ^b^higher score indicates higher function; ^c^higher scores indicate greater pain intensity; ^d^*P* < 0.001 ^e^*P* < 0.05

#### FAAM change

At the 6-week and 1-year follow-up, there was no between-group difference in the FAAM and both groups demonstrated significant improvement from baseline (Table 3 and Fig. 2). The proportion of participants that exceeded the MCID increased as the length of follow-up increased in both groups, but there was no between-group difference (Table 4 and Additional file 3).

**Table 4:** Proportion of participants that reported changes equal to or greater than the minimal clinically important difference (MCID) for pain and function ratings. The MCID for the numeric pain rating scale (NPRS) was a 2-point change and for the foot and ankle ability measure (FAAM) was an 8-point change. Values are number of participants (%) that reached or surpassed the MCID threshold or relative risk (95% CI) of achieving the MCID with usual podiatric care plus physical therapy treatment (uPOD+PT) versus usual podiatric care (uPOD) for the intention-to-treat (ITT) and the per-protocol (PP) analyses

	uPOD+PT	uPOD	Relative Risk (95% CI)
FAAM			
ITT analysis (uPOD+PT, *n* = 48; uPOD, *n* = 47)			
6 wk	36 (75)	31 (66)	1.1 (0.9 to 1.5)
6 mo	43 (89.6)	38 (80.9)	1.1 (0.9 to 1.3)
1 yr	44 (91.7)	42 (89.4)	1 (0.9 to 1.2)
PP analysis (uPOD+PT, *n* = 38; uPOD, *n* = 41)			
6 wk	29 (76.3)	26 (63.4)	1.2 (0.9 to 1.6)
6 mo	37 (97.4)	33 (80.5)	1.2 (1.0 to 1.4)^a^
1 yr	36 (94.7)	38 (92.7)	1 (0.9 to 1.1)
NPRS			
ITT analysis (uPOD+PT, *n* = 48; uPOD, *n* = 47)			
6 wk	31 (64.6)	16 (34)	1.9 (1.2 to 2.3)^a^
6 mo	32 (66.7)	27 (57.4)	1.2 (0.8 to 1.6)
1 yr	41 (85.4)	31 (66)	1.3 (1.0 to 1.6)^a^
PP analysis (uPOD+PT, *n* = 38; uPOD, *n* = 41)			
6 wk	26 (68.4)	15 (36.6)	1.9 (1.2 to 3.0)^a^
6 mo	30 (78.9)	23 (56.1)	1.4 (1.0 to 1.9)^a^
1 yr	34 (89.5)	28 (68.3)	1.3 (1.0 to 1.7)^a^

^a^*P* < 0.05

#### NPRS change

**Fig. 3:**
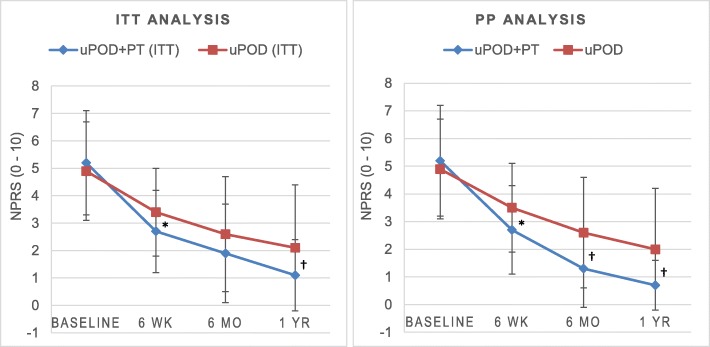
Numeric pain rating scale (NPRS) outcomes at each assessment for the primary intention-to-treat (ITT) and per-protocol (PP) analyses. Higher scores indicate greater pain intensity. *Change in NPRS from baseline significantly lower with usual podiatric care plus physical therapy treatment (uPOD+PT) versus usual podiatric care (uPOD) at *P* < 0.05 level or ^†^*P* < 0.001

There was no between-group difference in NPRS at 6 months, but the change in NPRS for the uPOD+PT group was greater than the uPOD group at the 6-week and 1-year follow-ups (Table 3 and Fig. 3). Both groups demonstrated significant improvement in NPRS change from baseline at each follow-up (Table 3). The proportion of participants that exceeded the MCID increased as the length of follow-up increased in both groups (Table 4 and Additional file 4). The proportion of participants in the uPOD+PT group that improved greater than the NPRS MCID exceeded the uPOD group by 31% at 6 weeks and 19% at 1 year, but there were no between-group differences at 6 months.

#### Global rating of change (GRC)

Patient-reported success increased for each group over time, but there were no between-group differences at any point (Table 5 and Additional file 5).

**Table 5:** Patient-reported success outcomes of treatment for plantar heel pain. Success was defined as ratings of “a great deal better” or “a very great deal better” on a 15-point Likert global rating of change (GRC) scale. Values are number of participants (%) with a successful outcome or relative risk (95% CI) of success with usual podiatric care plus physical therapy treatment (uPOD+PT) versus usual podiatric care (uPOD) for the intention-to-treat (ITT) and the per-protocol (PP) analyses

	uPOD+PT	uPOD	Relative Risk (95% CI)
ITT analysis (uPOD+PT, *n* = 48; uPOD, *n* = 47)			
6 wk	14 (29.2)	6 (12.8)	2.3 (1.0 to 5.4)
6 mo	29 (60.4)	26 (55.3)	1.1 (0.8 to 1.5)
1 yr	38 (79.2)	29 (61.7)	1.3 (1.0 to 1.7)
PP analysis (uPOD+PT, *n* = 38; uPOD, *n* = 41)			
6 wk	13 (34.2)	5 (12.2)	2.8 (1.1 to 7.1)^a^
6 mo	29 (76.3)	21 (51.2)	1.5 (1.1 to 2.1)^a^
1 yr	35 (92.1)	26 (63.4)	1.5 (1.1 to 1.9)^a^

^a^*P* < 0.05

### Per-protocol analyses

The PP analyses consisted of 38 participants allocated to receive uPOD+PT and 41 participants allocated to receive uPOD that completed treatment. Of the 10 participants that did not receive or complete uPOD+PT treatment, 4 did not attend a physical therapy appointment and 6 had fewer than 3 visits with a physical therapist; 1 stopped treatment with the physical therapist because of a worker’s compensation conflict, and 1 developed a separate problem that became the priority of physical therapy treatment.

#### Primary outcome

The uPOD+PT group had greater changes from baseline for the FAAM at 6 months (Table 3 and Fig. 2).

#### FAAM change

At the 6-week follow-up there was no between-group difference in the FAAM, but the uPOD+PT group had greater changes from baseline at 1 year (Table 3 and Fig. 2). The proportion of participants that exceeded the MCID increased as the length of follow-up increased in both groups (Table 4, Additional files 3). The proportion of participants in the uPOD+PT group that improved greater than the FAAM MCID exceeded the uPOD group by 17% at 6 months.

#### NPRS change

The uPOD+PT group had greater changes from baseline at every follow-up for the NPRS (Table 3, Fig. 3). The proportion of participants that exceeded the MCID increased as the length of follow-up increased in both groups, but the proportion of participants in the uPOD+PT group that improved greater than the NPRS MCID exceeded the uPOD group by 32% at 6 weeks, 23% at 6 months, and 21% at 1 year (Table 4, Additional files 4).

#### Global rating of change (GRC)

Patient-reported success increased for each group over time, but participants in the uPOD+PT group were 2.8 (1.1, 7.1), 1.5 (1.1, 2.1), and 1.5 (1.1, 1.9) times more likely than the uPOD group to report success at 6 weeks, 6 months, and 1 year, respectively (Table 5, Additional file 5).

## Discussion

In this randomized clinical trial, there was no significant benefit of uPOD+PT in the primary outcome of FAAM change at 6 months, although secondary outcomes and PP analysis indicated additional benefit of uPOD+PT, mostly observed in individuals who completed treatment. When between-group differences were observed, the additional benefit of physical therapy treatment to usual podiatric care was small to moderate in magnitude (eg, reduced NPRS by 0.9–1.5 points or improved FAAM by 5.5–7.7 points), although coincided with an increase in the proportion of participants achieving the MCID by 17–31%. Further research is needed to elucidate the importance of pain and functional improvements that considers the cost-effectiveness and the impact of uPOD or uPOD+PT on a person’s participation in domestic, work, and community, social and civic life.

Outcomes of the ITT analysis were provided alongside results from PP analysis to examine the influence of treatment compliance. Twenty-one percent (10/48) of participants allocated to uPOD+PT did not complete treatment compared to 13% (6/47) allocated to uPOD. The results of this trial including the lack of difference in the primary outcome via ITT analysis may be due no additional benefit of uPOD+PT, explained by underestimation of uPOD+PT treatment effect in the ITT analysis [61], or reflect inflation of the uPOD+PT effects in the PP analysis due to greater susceptibility to bias with PP versus ITT analysis [52]. Support for potential underestimation of uPOD+PT benefit is provided by the significant improvement with uPOD+PT observed in the PP analysis in the primary outcome and almost every other secondary variable. In addition, when comparing the full sample (ITT) to participants that completed treatment (PP), there was minimal change for the uPOD group (49%), but the percentage that did not achieve success with treatment dropped from 40 to 24% for the uPOD+PT group. A similar effect was observed at 1 year; the percentage that did not achieve success dropped from 21 to 8% in the uPOD+PT group compared to a drop from 38 to 37% in the uPOD group. The percentage that did not complete uPOD+PT (21%) is similar to the findings of Saban et al. [62], where 26% did not complete physical therapy treatment for PHP and Bassett et al. [54] where 17% did not complete physical therapy treatment for ankle sprain. Reasons for failure to complete treatment were not collected in this study, but lack of time, expense of treatment, feeling better, or forgetting to follow-up are reasons why patients did not complete treatment for ankle sprain [54]. The addition of physical therapy treatment to usual care required a median of 4 more visits with a provider in this study which demands more time and financial resources from the patient. Lack of time and concerns about treatment cost were the most frequent reasons observed by those who chose not to participate in this study (Fig. 1), and despite attempts to fully inform participants prior to participation, these concerns may have contributed to the failure to complete treatment. While it is possible that some participants did not complete treatment due to perceived lack of improvement, all 10 of the participants that did not complete treatment failed to see a physical therapist or had 3 or fewer visits to allow a response to be observed. Although further research is needed, any additional effect of physical therapy treatment to usual podiatric care appears to be improved when individuals complete their course of treatment.

Other explanations for the lack of difference in the primary outcome of this study may be the pragmatic design that resulted in 11 participants in the uPOD group receiving physical therapy treatment over the course of the study. Although, it is possible that this muted between-group contrasts, post-hoc subgroup analyses were avoided to due lack of power and the high risk of spurious findings [52]. Another consideration to explain the lack of difference is that uPOD+PT has a greater impact on certain patients, but less impact on others. Theoretically, the advantage of uPOD+PT is that physical therapists can perform manual therapy interventions, treat both local and proximal impairments contributing to PHP, and can facilitate return to higher level functions (eg, sports-related activities). Therefore, physical therapy treatment may be of greater benefit to those who have proximal impairments, are likely to benefit from manual therapy, or have goals related to return to sport-related activities. In this study, only the activities of daily living subscale, and not the sports subscale, of the FAAM was used to estimate change in function which may not capture the magnitude of change in patients functioning at a higher level. Also, while there is evidence supporting the additional benefit of manual therapy [51, 62], other factors to determine the benefit of adding physical therapy treatment are unknown to include predictors of response to manual therapy, the incidence of proximal factors contributing to PHP, and effectiveness of proximal interventions in PHP. Further investigation is required to determine unbiased estimates of uPOD+PT treatment effects including subgroups of persons that may benefit most from interventions provided by a physical therapist and less pragmatic designs that restrict participants in the comparison group from physical therapy treatment.

This is the first study to compare pragmatic, multimodal treatment provided by podiatrists and physical therapists for PHP, but the results are similar to other outcome studies of treatment for PHP. The majority of patients with PHP demonstrate significant improvement with non-surgical treatment 6 months to 1 year after starting treatment which is consistent with our results [24–26, 29, 51]. In addition, the outcome of the uPOD+PT group is comparable to a previous pragmatic trial of multimodal treatment for PHP that found a 31.6 point average improvement in FAAM and a 3.4 point average reduction in NPRS at the 6-month follow-up [51]. Similar to the current study, physical therapists in the Cleland et al. [51] study provided manual therapy and exercise interventions using an evidence-based clinical reasoning framework directed at foot, ankle, knee, and hip impairments. In the current study, physical therapy treatment also included lumbar and neurodynamic intervention only when supported by within- and between-session responses. The majority of physical therapy treatments included exercise interventions followed by manual therapy (Additional file 1) with limited use of modalities as supported by the results of Cleland et al. [51] and clinical practice guidelines [2]. Physical therapy treatment visits in the current study were less frequent and provided over a longer period of time than the 6 visits in 4 weeks used in the Cleland et al. [51] study. In addition, physical therapy treatment in the current study was guided by phases of rehabilitation and supplemented by patient education about pain neuroscience and factors related to their PHP recovery [49]. The results of this study and the study by Cleland et al. [51] demonstrate that pragmatic, impairment-based physical therapy treatment can provide meaningful improvements in pain and function 6-months after starting treatment. The current study also demonstrated additional benefit of physical therapy to podiatry treatment at 6 weeks and 1 year.

Meaningful changes in function and pain were achieved even though the average BMI of participants was at a level considered obese. Obesity is associated with PHP and diminished treatment response to conservative treatment [25, 63–65], but one study has demonstrated that obese individuals can achieve a successful response to physical therapy treatment [42]. Specific weight management strategies were not implemented in this study, but weight management was promoted by goals focused on increasing activity levels and returning to regular exercise [49]. No evidence was found that demonstrated the effect of weight loss through diet and/or exercise on outcomes in individuals PHP or other foot conditions, but evidence for weight loss through bariatric surgery exists. An observational study found that 90% of obese individuals that reduced their BMI by an average of 10.2 kg/m^2^ after bariatric surgery required less treatment visits and modalities for their PHP [66]. In addition, 2 other studies of patients with foot pain not specific to plantar heel pain, support improvement in foot function or pain following bariatric surgery [67, 68]. Despite limited randomized clinical trials to determine efficacy, clinical guidelines recommend weight loss strategies within the management of PHP and these strategies may improve the outcomes achieved in this study [2]. Similar to other studies [25–29], we found that full patient-perceived recovery was not achieved in all participants despite overall improvement. While patient-perceived recovery is assessed differently, we used the top two ratings for GRC as an indicator of recovery/successful outcome. Forty percent of participants in the uPOD+PT and 45% in the uPOD group did not achieve success at the 6-month follow-up.

Ten participants (10.5%) were referred to, and attended a visit with a physical therapist within 30 days of the initial visit with the podiatrist which is slightly higher than the 7.5% rate of physical therapy utilization observed by Fraser et al. [40]. Because participants were informed of the study to include a treatment arm that may include physical therapy treatment, this rate of referral may be inflated by provider and patient bias due to increased awareness of physical therapy as a treatment option. The low referral rate to a physical therapist may be due to evidence that most patients improve with conservative treatment [24, 25, 69], and a lack of evidence to support the additional effect of physical therapy treatment to usual care. But, delayed referral to physical therapy has consequences and patients with PHP symptoms greater than 7 months are 4 times less likely to have a successful response to physical therapy treatment [42]. In the uPOD group, 4 participants were immediately referred to a physical therapist, while 7 participants were referred from 19 to 169 days after the first visit with the podiatrist. When appropriate, prompt referral to a physical therapist would appear to enhance the likelihood of a good outcome and significant delays may hinder the patient’s recovery potential.

### Limitations

Generalizability of this investigation is limited because it was conducted at one clinic by a limited number of providers. The usual practice patterns and interdisciplinary collaboration within this study may not represent practices in other health systems or countries. The physical therapy and podiatry interventions used in this study may not be discipline-specific, but provider-specific terms (podiatrist and physical therapist) were used throughout this study to clearly depict the providers of the interventions. Physical therapy and podiatry practice patterns were consistent with American-based professional associations’ practice guidelines [2, 12]. Further research of interdisciplinary treatment approaches in other health systems and regions is needed, however the outcomes of uPOD+PT group were similar to those achieved in a separate investigation that included 6 physical therapists from 2 different clinics in 2 different countries [51]. All participants were seeking care from a podiatrist and the effects of the uPOD+PT group may have been affected by allocation to treatment other than uPOD, although there is limited evidence that patient preference substantially affects internal validity [70]. Estimates derived from PP analysis are more prone to bias than ITT analysis and should be interpreted with caution [52].

The pragmatic design of this study limits conclusions regarding specific interventions (e.g. manual therapy, stretching or strengthening exercises, injections, etc), and precise duplication of specific interventions. Because treatment was multimodal and pragmatic, further research is needed to elucidate the mechanisms of specific interventions and to identify factors that predict patient response to specific treatments or treatment approaches. To assist in duplication of the physical therapy intervention, further clinical reasoning details and intervention descriptions are provided by McClinton et al. [49]. In addition, the pragmatic design that reflects typical clinical patterns resulted in uPOD+PT participants having a median of 4 more visits than the uPOD group and therefore between-group changes may be due to an attention effect.

## Conclusions

The ITT analysis indicated no additional benefit of uPOD+PT to uPOD in the primary outcome of FAAM change at 6 months. Secondary outcomes from ITT analysis demonstrated improvements in pain at 6 weeks and 1 year that favored the uPOD+PT group. Per-protocol analysis demonstrated additional benefit and a higher rate of success of uPOD+PT, but due to potential bias of PP analysis, these findings requires more rigorous investigation.

## Supplementary information

**Additional file 1.** Details of treatment provided to the usual podiatric care (uPOD) and usual podiatric care plus physical therapy treatment (uPOD + PT) groups. Table including foot orthoses, medication, and treatment procedures provided to both treatment groups.

**Additional file 2.** Format: Treatment adherence in the usual podiatric care (uPOD) and usual podiatric care plus physical therapy treatment (uPOD + PT) groups. Table of treatment adherence rankings for each treatment group.

**Additional file 3.** Percentage of participants that reported changes equal to or greater than the minimal clinically important difference (MCID) of eight points for the foot and ankle ability measure (FAAM) for the intention-to-treat (ITT) and per-protocol (PP) analyses. Bar graphs of the percentage of patients that reported changes in FAAM that exceeded the MCID.

**Additional file 4.** Percentage of participants that reported changes equal to or greater than the minimal clinically important difference (MCID) of two points for the numeric pain rating scale (NPRS) for the intention-to-treat (ITT) and per-protocol (PP) analyses. Bar graphs of the percentage of patients that reported changes in NPRS that exceeded the MCID.

**Additional file 5.** Treatment success outcomes at each assessment for the intention-to-treat (ITT) and per-protocol (PP) analyses. Bar graphs of the percentage of patients that reported treatment success based on the GRC.

## Data Availability

The datasets used and/or analyzed during the current study are available from the corresponding author on reasonable request.

## Abbreviations

BMI: Body mass
FAAM: Foot and Ankle Ability Measure
GRC: Global rating of change scale
ITT: Intention-to-treat
mo: month
NPRS: Numeric pain rating scale
PHP: Plantar heel pain
PP: Per-protocol
PT: Physical therapist
uPOD: usual podiatric care
uPOD+PT: physical therapy treatment in addition to usual podiatric care
wk: week
yr: year
