# Supplementary material for: Effectiveness of physical therapy treatment in addition to usual podiatry management of plantar heel pain: a randomized clinical trial
Source: BMC Musculoskelet Disord. 2019 Dec 28;20:630. doi: 10.1186/s12891-019-3009-y (PMC6935140; doi:10.1186/s12891-019-3009-y)
Supplement: Supplementary file 1 — Additional file 1. Details of treatment provided to the usual podiatric care (uPOD) and usual podiatric care plus physical therapy treatment (uPOD + PT) groups. Table including foot orthoses, medication, and treatment procedures provided to both treatment groups. [file 12891_2019_3009_MOESM1_ESM.docx]

**Additional file 1. Details of treatment provided to the usual podiatric care (uPOD) and usual podiatric care plus physical therapy treatment (uPOD+PT) groups.** Values are frequency count (%) unless otherwise indicated.

| Treatment detail | uPOD+PT (n=48) | uPOD (n=47) |
| --- | --- | --- |
| Foot orthosis prescribed by podiatrist^†^ | | |
| None, or modified current OTC or custom orthosis | 18 (38) | 19 (40) |
| Spenco RX^®‡^: Hard, non-posted moulded, ¾ length, soft topcover | 6 (12) | 6 (13) |
| ProLab P3^§^: Hard, posted moulded, full length, soft topcover | 8 (17) | 11 (24) |
| JM Orthotics^‖^: Hard, non-posted moulded, ¾ length, no topcover | 12 (25) | 7 (15) |
| Spenco Cushion^‡^: Soft, non-posted non-moulded, ¾ length, soft topcover | 2 (4) | 3 (6) |
| Custom: Hard, posted, moulded, full length with soft topcover | 2 (4) | 1 (2) |
| Medication prescribed by podiatrist | | |
| None | 16 (33) | 19 (38) |
| OTC NSAID | 20 (42) | 14 (30) |
| Diclofenac Sodium (with or without misoprostol) | 7 (15) | 6 (13) |
| Methylprednisolone | 6 (13) | 6 (13) |
| Meloxicam | 4 (8) | 3 (6) |
| Naproxen | 1 (2) | 2 (4) |
| Podiatry-specific procedures: total procedural units billed (%) patients that received intervention) | | |
| New evaluation | 44 (92) | 45 (96) |
| Established visit | 40 (42) | 56 (70)* |
| Injection | 17 (19) | 18 (19) |
| Radiographs | 5 (10) | 5 (6) |
| MRI | 2^††^ (4) | 1 (2) |
| Surgery | 1^††^ (2) | 1 (2) |
| Post-operative evaluation | 4^††^ (2) | 3 (2) |
| Physical Therapy-specific procedures^#^: total procedural units billed (%) patients that received intervention | | |
| New evaluation | 44 (92) | 9 (90) |
| Therapeutic exercise | 257 (92) | 82 (90) |
| Manual therapy | 152 (85) | 68 (70)* |
| Therapeutic activity | 28 (38) | 4 (20) |
| Neuromuscular re-education | 5 (8) | 9 (20)* |
| Ultrasound | 0 (0) | 11 (20)* |
| Iontophoresis | 3 (2) | 0 (0) |
| Physical performance test (gait analysis) | 2 (4) | 1 (10) |
| Re-evaluation | 1 (2) | 0 (0) |

OTC = over-the-counter; NSAID = non-steroidal anti-inflammatory drug; PT = physical therapist. *Significantly different from the uPOD+PT group, *P* < .05; ^†^Hard material was classified as a Shore A durometer rating of 93-99; soft was 23-51. Posting and moulding categories as defined by Mills et al (70); ^‡^Spenco, Waco, TX; ^§^ProLab Orthotics, Napa, CA; ^‖^JM Orthotics, St. George, UT; ^#^For the uPOD group, n=10; ^††^Participants did not initiate or did not complete physical therapy treatment.
