# Supplementary material for: Effectiveness of physical therapy treatment in addition to usual podiatry management of plantar heel pain: a randomized clinical trial
Source: BMC Musculoskelet Disord. 2019 Dec 28;20:630. doi: 10.1186/s12891-019-3009-y (PMC6935140; doi:10.1186/s12891-019-3009-y)
Supplement: Supplementary file 2 — Additional file 2. Format: Treatment adherence in the usual podiatric care (uPOD) and usual podiatric care plus physical therapy treatment (uPOD + PT) groups. Table of treatment adherence rankings for each treatment group. [file 12891_2019_3009_MOESM2_ESM.docx]

**Additional file 2. Treatment adherence in the usual podiatric care (uPOD) and usual podiatric care plus physical therapy treatment (uPOD+PT) groups.** Adherence was rated on a numeric rating scale where 0=no treatment completed, and 10=completed all treatment as instructed. Values are median (IQR).

| Treatment detail | uPOD+PT (n=48) | | | uPOD (n=47) | | |
| --- | --- | --- | --- | --- | --- | --- |
| Treatment Adherence | 6-week | 6-month | 1-year | 6-week | 6-month | 1-year |
| Medication | 5 (0-9) | 7 (0-10) | 8.8 (1.3-10) | 8 (0-10) | 8 (2.5-10) | 8 (6-10) |
| Foot Orthosis | 8 (4.3-10) | 8 (5-9.8) | 8.8 (5.3-10) | 9.6 (7-10) | 9 (7.3-10) | 9.5 (8-10) |
| Exercises from podiatrist^†^ | 7 (5-8) | NA | NA | 8 (6-8.6) | 7 (5-8.7) | 8 (5-9) |
| Exercises from PT^‡^ | 9 (7.1-9) | 8.3 (7-9.5) | 9 (7-9.6) | 7 (1.8-8.5)* | 8.8 (7-9) | 8.3 (7-9) |
| Footwear modification | 8 (2.5-10) | 9 (6.5-10) | 9 (7.8-10) | 8 (4-10) | 9 (5.8-10) | 9 (7.8-10) |
| Activity modification | 5 (0-9) | 8 (5.8-9.6) | 9 (3-9.5) | 0 (0-8) | 7 (0-10) | 5 (0-9) |

PT = physical therapist. *Significantly different from the uPOD+PT group, *P* < .05; ^†^For the uPOD+PT group, exercises from podiatrist only performed during the time between the initial evaluation by podiatrist and the first PT visit and therefore 6-month adherence is not applicable. ^‡^For the uPOD group, n=10.
