# Supplementary material for: Effectiveness of physical therapy treatment in addition to usual podiatry management of plantar heel pain: a randomized clinical trial
Source: BMC Musculoskelet Disord. 2019 Dec 28;20:630. doi: 10.1186/s12891-019-3009-y (PMC6935140; doi:10.1186/s12891-019-3009-y)
Supplement: Supplementary file 3 — Additional file 3. Percentage of participants that reported changes equal to or greater than the minimal clinically important difference (MCID) of eight points for the foot and ankle ability measure (FAAM) for the intention-to-treat (ITT) and per-protocol (PP) analyses. Bar graphs of the percentage of patients that reported changes in FAAM that exceeded the MCID. [file 12891_2019_3009_MOESM3_ESM.docx]

**Additional file 3. Percentage of participants that reported changes equal to or greater than the minimal clinically important difference (MCID) of eight points for the foot and ankle ability measure (FAAM) for the intention-to-treat (ITT) and per-protocol (PP) analyses.** *Significant difference in proportion of participants that met or exceeded the MCID with usual podiatric care plus physical therapy treatment (uPOD+PT) versus usual podiatric care (uPOD) (*P* < 0.05).
