# Supplementary material for: Effectiveness of physical therapy treatment in addition to usual podiatry management of plantar heel pain: a randomized clinical trial
Source: BMC Musculoskelet Disord. 2019 Dec 28;20:630. doi: 10.1186/s12891-019-3009-y (PMC6935140; doi:10.1186/s12891-019-3009-y)
Supplement: Supplementary file 5 — Additional file 5. Treatment success outcomes at each assessment for the intention-to-treat (ITT) and per-protocol (PP) analyses. Bar graphs of the percentage of patients that reported treatment success based on the GRC. [file 12891_2019_3009_MOESM5_ESM.docx]

Additional file 5. **Treatment success outcomes at each assessment for the intention-to-treat (ITT) and per-protocol (PP) analyses.** Success was defined as ratings of “a great deal better” or “a very great deal better” on a 15-point Likert global rating of change scale. *Significant difference in relative risk of success with usual podiatric care plus physical therapy treatment (uPOD+PT) versus usual podiatric care (uPOD) (*P* < 0.05)
